# Ovarian Endometrioma Negatively Impacts Oocyte Quality and Quantity But Not Pregnancy Outcomes in Women Undergoing IVF/ICSI Treatment: A Retrospective Cohort Study

**DOI:** 10.3389/fendo.2021.739228

**Published:** 2021-11-22

**Authors:** Yaoqiu Wu, Rong Yang, Jie Lan, Haiyan Lin, Xuedan Jiao, Qingxue Zhang

**Affiliations:** Reproductive Medicine Center, Sun Yat-sen Memorial Hospital, Sun Yat-sen University, Guangzhou, China

**Keywords:** endometrioma, oocyte quality and competence, ovarian response, *in vitro* fertilization/intracytoplasmic sperm injection and frozen–thawed embryo transfer, ovarian surgery

## Abstract

**Purpose:**

To determine the impact of ovarian endometrioma *per se* on *in vitro* fertilization/intracytoplasmic sperm injection (IVF/ICSI) outcomes.

**Methods:**

This retrospective study was conducted using two groups. The endometrioma group consisted of 862 women with infertility who had ovarian endometriomas and underwent their first ovarian stimulation for IVF/ICSI treatment between January 2011 to December 2019 at a public university hospital. A non-endometrioma comparison group, comprising 862 women with other infertility factors, was matched according to maternal age, body mass index (BMI), and infertility duration. Ovarian reserve and response and IVF/ICSI and pregnancy outcomes between the two groups were analyzed. Multivariate logistic regression (MLR) analysis was conducted on the basis of clinical covariates assessed for their association with live birth.

**Results:**

The results showed that significantly lower antral follicle count (AFC), anti-Müllerian hormone (AMH), ovarian sensitivity index (OSI), oocyte maturation and fertilization rates, blastocyst rate, number of oocytes retrieved, and available embryos were found in women with endometrioma compared with the control, respectively (P < 0.05). The cumulative live birth rate *per* patient in women with endometrioma was lower than that of women without endometrioma (39.32% *vs*. 46.87%, P = 0.002). In women with endometrioma, those who underwent surgical intervention prior to IVF/ICSI treatment had higher maturation (86.03% *vs*. 83.42%, P = 0.003), fertilization (78.16% *vs*. 74.93%, P = 0.004), and top-quality embryo rates (42.94% *vs*. 39.93%, P = 0.097) but had fewer oocytes retrieved (8.01 ± 5.70 *vs*. 9.12 ± 6.69, P = 0.013) than women without surgery. However, live birth rates were comparable between women with endometrioma and women in the control group, regardless of whether they had a prior history of ovarian surgery. MLR analysis showed no correlation between endometrioma *per se* and live birth after being adjusted for number of top-quality embryos transferred and stage of embryo transfer.

**Conclusions:**

The data from this study supported the conclusion that ovarian endometrioma negatively impacts oocyte quality and quantity, but not overall pregnancy outcomes, in women undergoing IVF/ICSI treatment. Endometrioma lowers the cumulative live birth rate by decreasing the number of embryos. Surgical excision of endometrioma prior to IVF/ICSI can partly improve oocyte maturation and fertilization rates but not pregnancy outcomes.

## Introduction

Endometriosis (EMS) is a common, benign disease characterized by the histological presence of functional endometrial glands or stroma outside the uterine cavity, affecting about 50% of infertile women (1). Ovarian endometrioma is one of the most common forms of EMS, present in up to 30%–40% of women with EMS (1). This condition is characterized by the presence of one or more cysts stemming from endometrial tissue that is histologically and functionally similar to eutopic endometrium (2).

The pathogenesis of EMS-associated infertility is poorly elucidated. In particular, the impact of endometriomas on ovarian function has yet to be clearly established. Some studies have reported that endometriomas negatively impact oocyte quality and quantity by increasing mechanical stress, thus destroying the normal anatomy to impair blood supply and innervation (2–4). Other studies have revealed that local inflammation and toxic content such as free iron diffusing from the endometrioma cyst into nearby ovarian tissue result in oocyte loss and poor embryo quality (5–8). Besides, Kitajima et al. reported that ovaries with endometriomas showed activated follicular recruitment and atresia of early follicles (9). Still, one study held the point that endometrioma *per se* is not associated with infertility (10). Surgical interventions are sometimes performed in women with endometrioma, but reduced ovarian reserves and responsiveness, as well as postoperative recidivism, have been noted by researchers studying such women (11–14). Pregnancy results after *in vitro* fertilization (IVF) or intracytoplasmic sperm injection (ICSI) treatment were not improved in women with surgical treatment of endometriomas smaller than 6 cm compared to the women who refused operation (15). Thus, some uncertainty remains regarding the benefits of surgery to remove cysts before pregnancy.

Assisted reproductive technology (ART), including IVF/ICSI, is extensively used to manage EMS-related infertility. However, the influence of endometrioma *per se* on ART outcomes is still undetermined. ART results vary by report, with some showing decreased numbers of oocytes retrieved and poor embryo quality, and other results present identical live birth rates as in patients without endometrioma despite fewer oocytes retrieved during IVF treatment (16). Previous studies have mostly focused on the effect of surgical removal of endometrioma on ART outcomes rather than the effect of the endometrioma itself or have included a single-endometrioma group without a matching control group for comparison (17, 18).

Therefore, this retrospective study was conducted in those women with endometrioma, accompanied with a matching non-endometrioma control group, to determine the influence of endometrioma on reproductive outcomes.

## Patients and Methods

### Data Sources

Infertile women presenting one or more endometriomas (at least one ovarian endometrioma of ≥15 mm and with no other infertility factors) in unilateral or bilateral ovaries before or after ovarian surgery, who also underwent their first fresh or frozen IVF/ICSI cycles with autologous oocytes between 2011 and 2019 at Sun Yat-sen Memorial Hospital in Guangzhou, China, were screened for this retrospective cohort study. Endometrioma diagnoses were made by expert sonographers using transvaginal ultrasonography (19) or magnetic resonance imaging or following abdominopelvic surgery. Women with endometrioma who had surgical intervention experienced laparoscopic ovarian cystectomy for cyst removal prior to IVF/ICSI treatment. The exclusion criteria were as follows: polycystic ovarian syndrome; adenomyosis; systemic lupus erythematosus or another rheumatologic disease; or use of hormonal medications or hormonal or non-hormonal anti-inflammatory agents during the 3 months prior to inclusion in this study. For comparison, women without any ovarian cysts who underwent ovarian stimulation for IVF/ICSI due to tubal factor or incretion factor other than EMS during the same time period were randomly matched for maternal age, body mass index (BMI), and infertility duration with the endometrioma group. All patients underwent a baseline fertility assessment prior to IVF that included basal day 3 follicle-stimulating hormone (FSH) and anti-Müllerian hormone (AMH) level, as well as a pelvic ultrasound for antral follicle count (AFC). All clinical records/information was anonymized and deidentified prior to analysis.

### *In Vitro* Fertilization/Intracytoplasmic Sperm Injection Treatment Procedure

All ovarian stimulation cycles included in this study were first IVF/ICSI attempts for all patients, who underwent conventional stimulation following a flexible gonadotropin-releasing hormone (GnRH) agonist or an antagonist protocol, according to the observed follicular growth by ultrasound and blood tests. Follicle growth was regularly monitored by transvaginal ultrasound, in addition to the serum estradiol (E2), progesterone, and luteinizing hormone (LH) levels during the cycle. Final oocyte maturation was induced by administering human chorionic gonadotropin (hCG) when at least one follicle exceeded 18 mm in diameter on ultrasonography. Oocyte aspiration was performed 34–36 h after hCG administration. Fresh embryo transfers were performed on either Day 3 or 5. Frozen–thawed transfer was performed through an ovulation induction cycle with gonadotropins or through a hormone replacement therapy (HRT) cycle with endometrial preparation by exogenous estrogen and progesterone or through the cycle adding GnRH-a before estradiol. No more than three embryos were transferred. The luteal supported phase was administered by vaginal administration of micronized progesterone (600 mg/day). Pregnancies were diagnosed by an increasing concentration of serum β-hCG, which was tested at 14 days after embryo transfer. Clinical pregnancies were confirmed by the presence of a gestational sac on vaginal ultrasound examination during the fifth week after embryo transfer. A live birth was defined as any birth event in which at least one baby was born alive.

The number of oocytes, metaphase stage II (MII) oocytes retrieved, total number of units of gonadotrophins used (IU), duration of ovarian stimulation (days), estradiol peak (pg/ml), number of follicles ≥14 mm on the day of trigger, fertilization rate, implantation rate, clinical pregnancy rate, live birth rate (LBR), and cumulative live birth rate (CLBR) were recorded and compared. The ovarian sensitivity index (OSI) (number of oocytes retrieved × 1,000/total dose of gonadotrophins administered) was calculated as described in previous publications (20). It has previously been suggested that OSI may be considered a better response marker to gonadotrophins than the number of oocytes obtained (21). To evaluate the effect of surgery on IVF/ICSI outcomes, the endometrioma women were further divided into two subgroups according to their history of ovarian surgery, and the relative clinical variables were analyzed and compared.

### Statistical Analysis

Data were obtained from the hospital information system, and statistical analyses were performed using the STATA software version 14 MP. Random matching was used to select the control cohort. Statistics that had a Gaussian distribution were presented as mean ± SD when categorical variables were described as absolute frequencies. The Spearman rank correlation test was used to identify the correlation between the OSI and regimen of ovarian hyperstimulation. Logistic regression analysis was conducted on clinical covariates assessed for their association with live birth. Multivariable logistic regression (MLR) analysis was performed on variables that were significant at univariable analysis (P < 0.05). A two-sided P value of less than 0.05 was considered significant.

## Results

### Endometrioma Lowers Ovarian Reserve

A total of 1,724 women were enrolled, including 862 women with endometrioma and 862 women without endometrioma. No significant differences were observed between the two groups in regard to age, BMI, and infertility duration, showing a valid matching in the enrolled population. The basal characteristics of the study participants are described in **Table 1**. Compared to women in the non-endometrioma group, women in the endometrioma group showed significantly lower AMH levels (3.02 ± 3.04 ng/ml *vs*. 5.74 ± 4.53 ng/ml, P < 0.001) and AFC (10.95 ± 7.22 *vs*. 17.74 ± 10.57, P < 0.001), accompanied by higher levels of FSH (9.69 ± 5.68 IU/L *vs*. 8.01 ± 3.60 IU/L). The other clinical features between the two groups showed no significant differences (**Table 1**).

**Table 1:** Demographic and clinical features of patients with and without endometrioma.

Characteristics^a^	Endometrioma (n = 862)	Non-Endometrioma (n = 862)	P
Maternal age (years)	32.73 ± 4.51	32.82 ± 4.82	0.683
Infertility duration (years)	4.16 ± 3.10	4.11 ± 3.08	0.749
BMI (kg/m^2^)	21.00 ± 5.53	21.43 ± 9.15	0.230
FSH (IU/L)	9.69 ± 5.68	8.01 ± 3.60	<0.001*
AMH (ng/ml)	3.02 ± 3.04	5.74 ± 4.53	<0.001*
AFC (n)	10.95 ± 7.22	17.74 ± 10.57	<0.001*
Endometrioma side			
Left n (%)	244 (28.31)		
Right n (%)	428 (49.65)		
Bilateral, n (%)	190 (22.04)		
Endometrioma size (mm)	21.61 ± 9.70		
Regimen of ovarian hyperstimulation			<0.001*
Ultra-long protocol	382 (44.32)	167 (19.37)	
Long protocol	346 (40.14)	409 (47.45)	
GnRH antagonists	134 (15.54)	286 (33.18)	
Duration of stimulation (days)	11.42 ± 3.48	10.76 ± 3.06	0.028*
Total dose of FSH administered (IU)	2,497.74 ± 996.52	1,984.64 ± 825.97	<0.001*
E2 at the time of hCG (pg/mL)	2,166.48 ± 1,439.52	2,198.77 ± 1,148.20	0.644
P at the time of hCG (pg/ml)	1.11 ± 0.66	1.08 ± 0.97	0.489

BMI, body mass index; AMH, anti-Müllerian hormone; AFC, antral follicle count; FSH, follicle-stimulating hormone; E2, estrogen; GnRH, gonadotropin-releasing hormone; hCG, human chorionic gonadotropin; P, progesterone. ^a^ Continuous variables are expressed as mean ± standard deviation, SD, categorical variables as absolute frequencies, n (%). *P < 0.05 was considered statistically significant.

### Endometrioma Adversely Impacts the Ovarian Response and Oocyte Competence but Not Pregnancy Outcomes

Women with endometrioma tended to choose ultra-long (44.32%) and long (40.14%) protocols, whereas women without endometrioma were prone to choosing long (47.45%) and GnRH antagonists (33.18%) protocols (P < 0.001) (**Table 1**). Endometrioma women experienced longer durations of stimulation and greater doses of FSH administered but a lower number of large follicles (13.73 ± 9.20 *vs*. 19.97 ± 12.17, P <0.001), oocytes retrieved (8.39 ± 6.07 *vs*. 11.75 ± 7.10, P <0.001), and mature oocytes (6.94 ± 5.33 *vs*. 9.50 ± 6.34, P < 0.001) (**Tables 1**, **2**). The OSI was significantly lower in endometrioma women than those in the non-endometrioma group (4.25 ± 4.27 *vs*. 7.40 ± 6.08, P < 0.001), showing a poor ovarian response among women with endometrioma. The Spearman rank correlation test implied that there was no significant correlation between OSI and regimen of ovarian hyperstimulation (ρ = 0.170, P = 0.105). The number of normal fertilized oocytes was lower in women with endometrioma compared to the control women, but no difference was found in the fertilization rate between the two groups. Besides, the blastocyst rate (49.36% *vs*. 65.32%, P < 0.001) and numbers of available embryos (3.88 ± 3.18 *vs*. 4.39 ± 3.12, P = 0.001) and top-quality embryos (1.54 ± 2.01 *vs*. 2.70 ± 2.72, P < 0.001) were lower in women with endometrioma when compared to the control group. The CLBR per patient was lower in women with endometrioma (39.32% *vs*. 46.87%, P = 0.002), but cycle cancellation rate, clinical pregnancy rate, ectopic pregnancy rate, and LBR per oocyte pickup cycle showed no significant differences between the two groups. The proportions of top-quality embryos and available embryos did not differ significantly between the two groups. Endometrioma *per se* showed a correlation with live birth in the results of univariate analysis (odds ratio, 0.592; 95% CI, 0.402–0.874; P = 0.043). However, MLR showed no correlation between endometrioma *per se* and live birth after being adjusted for number of top-quality embryos transferred and stage of embryo transfer (**Table 3**).

**Table 2:** Characteristics of IVF/ICSI outcomes in the studied population.

Characteristics^a^	Endometrioma (n = 862)	Non-Endometrioma (n = 862)	P
No. of follicles ≥ 14 mm	13.73 ± 9.20	19.97 ± 12.17	<0.001*
Number of oocytes retrieved	8.39 ± 6.07	11.75 ± 7.10	<0.001*
OSI	4.25 ± 4.27	7.40 ± 6.08	<0.001*
No. of MII	6.94 ± 5.33	9.50 ± 6.34	<0.001*
Maturation rate n (%)	82.78 (5,760/6,958)	84.71 (8,169/9,643)	0.001
No. of 2PN	5.23 ± 3.98	7.43 ± 4.98	<0.001*
Fertilization rate n (%)	76.96 (4,587/5,960)	78.15 (6,334/8,105)	0.095
No. of available embryos	3.88 ± 3.18	4.39 ± 3.12	0.001*
No. of top-quality embryos	1.54 ± 2.01	2.70 ± 2.72	<0.001*
Blastocyst rate, (%)	49.36 (656/1,329)	65.32 (1,946/2,979)	<0.001*
Cycle cancellation rate (%)	3.94 (34/862)	3.13 (27/862)	0.361
Stage of embryo transfer			<0.001*
Cleavage, n	490	668	
Blastocyst, n	338	167	
Type of embryo transfer			0.095
Fresh ET	601	575	
Frozen–thawed ET	227	260	
No. of top-quality embryos transferred (n)	1.10 ± 0.76	0.79 ± 0.80	<0.001*
Clinical pregnancy rate (%)	49.88 (413/828)	50.30 (420/835)	0.864
Ectopic pregnancy rate (%)	1.94 (8/413)	3.33 (14/420)	0.209
Miscarriage rate (%)	14.77 (61/413)	15.00 (63/420)	0.926
Live birth rate per cycle (%)	37.08 (307/828)	39.88 (333/835)	0.240
Cumulative live birth rate/patient (%)	39.32 (339/862)	46.87 (404/862)	0.002

FSH, follicle-stimulating hormone; E2, estrogen; P, progesterone; OSI, ovarian sensitivity index; MII, metaphase ii; PN, pronucleus; ET, embyo transfer. ^a^ Continuous variable are expressed as mean ± standard deviation, SD, categorical variables as absolute frequencies, n (%). *P < 0.05 was considered statistically significant.

**Table 3:** Univariate and multivariate analysis of factors affecting the live birth in patients undergoing IVF/ICSI.

	Univariate analysis		Multivariate analysis	
	OR 95% CI	P value	OR 95% CI	P value
Age	1.023 (0.979–1.069)	0.312		
BMI	0.951 (0.885–1.022)	0.173		
AMH	1.041 (0.979–1.107)	0.200		
Endometrioma *per se*	0.592 (0.402–0.874)	0.043*	0.680 (0.527–1.878)	0.108
Regimen of ovarian hyperstimulation				
Ultra-long protocol	Reference	0.159		
Long protocol	1.386 (0.749–2.564)			
GnRH antagonists	0.870 (0.562–1.346)			
OSI	0.897 (0.522–1.543)	0.569		
No. of top-quality embryo transferred	1.298 (1.027–1.623)	0.023*	1.267 (1.019–1.576)	0.034*
Stage of embryo transfer				
Cleavage	Reference			
Blastocyst	1.004 (1.000–1.008)	0.061	1.082 (0.624–1.875)	0.779
Type of embryo transfer				
Fresh ET	Reference			
Frozen-thawed ET	0.869 (0.580–1.301)	0.495		

BMI, body mass index; AMH, anti-Müllerian hormone; OSI, ovarian sensitivity index; GnRH, gonadotropin-releasing hormone; ET, embryo transfer. *P < 0.05 was considered statistically significant.

### Surgery Prior to *In Vitro* Fertilization/Intracytoplasmic Sperm Injection Treatment Improved Oocyte and Embryo Quality in Women With Endometrioma

To explore the impact of ovarian surgery on IVF/ICSI outcomes, the women with endometrioma were divided into two subgroups according to prior history of ovarian surgery. A total of 569 women had experienced ovarian surgery, while the remaining 293 women had not before IVF/ICSI treatment. The characteristics of IVF/ICSI in the two subgroups are shown in **Table 4**. The basal clinical features, including age, BMI, basal E2, and FSH, showed no significant differences between the two subgroups. AMH (2.88 ± 3.09 *vs*. 3.24 ± 2.97), AFC (9.78 ± 7.24 *vs*. 11.31 ± 7.18), and OSI (4.52 ± 4.35 *vs*. 4.11 ± 4.23) were lower in endometrioma women with surgery than in women without surgery, but no significant difference was found. Women with surgery had a lower number of large follicles (12.94 ± 8.77 *vs*. 15.26 ± 9.83, P = 0.001), oocytes retrieved (8.01 ± 5.70 *vs*. 9.12 ± 6.69, P = 0.013), and mature oocytes (6.63 ± 5.07 *vs*. 7.54 ± 5.77, P = 0.017) but higher maturation (86.03% *vs*. 83.42%, P = 0.003), fertilization (78.16% *vs*. 74.93%, P = 0.004), and top-quality embryo rates (42.94% *vs*. 39.93%, P = 0.097) than those in women without surgery.

**Table 4:** Characteristics of IVF/ICSI in endometrioma women with and without surgery.

Characteristics^a^	Endometrioma with surgery (n = 569)	Endometrioma without surgery (n = 293)	P
Maternal age (years)	32.63 ± 4.54	32.91 ± 4.49	0.385
Infertility duration (years)	4.20 ± 3.00	4.09 ± 3.29	0.628
BMI (kg/m^2^)	21.15 ± 6.59	20.71 ± 2.36	0.267
AFC (n)	9.78 ± 7.24	11.31 ± 7.18	0.211
AMH (ng/ml)	2.88 ± 3.09	3.24 ± 2.97	0.094
Regimen of ovarian hyperstimulation			0.090
Ultra-long protocol	41.30 (235/569)	50.17 (147/293)	
Long protocol	42.18 (240/569)	36.17 (106/293)	
GnRH antagonists	9.14 (52/569)	6.83 (20/293)	
Others	7.38 (42/569)	6.83 (20/293)	
No. of follicles ≥14 mm (n)	12.94 ± 8.77	15.26 ± 9.83	0.001*
No. of oocytes retrieved (n)	8.01 ± 5.70	9.12 ± 6.69	0.013*
OSI	4.52 ± 4.35	4.11 ± 4.23	0.199
No. of MII (n)	6.63 ± 5.07	7.54 ± 5.77	0.017*
Maturation rate (%)	86.03 (3,750/4,359)	83.42 (2,210/2,649)	0.003
No. of 2PN (n)	5.09 ± 3.86	5.51 ± 4.19	0.146
Fertilization rate (%)	78.16 (2,931/3,750)	74.93 (1,656/2,210)	0.004*
No. of available embryos	3.82 ± 3.18	4.01 ± 3.17	0.408
No. of top-quality embryos	1.52 ± 2.01	1.56 ± 2.00	0.781
Top-quality embryos rate (%)	42.94 (892/2,077)	39.93 (458/1,147)	0.097

BMI, body mass index; AMH, anti-Müllerian hormone; AFC, antral follicle count; OSI, ovarian sensitivity index; GnRH, gonadotropin-releasing hormone; MII, metaphase ii; PN, pronucleus. ^a^Continuous variable are expressed as mean ± standard deviation, SD, categorical variables as absolute frequencies, n (%). *P < 0.05 was considered statistically significant.

### Surgery Prior to *In Vitro* Fertilization/Intracytoplasmic Sperm Injection Treatment Did Not Impact Pregnancy Outcomes

The pregnancy outcomes of the two subgroups are shown in **Table 5**. In the surgery group, a total of 401 women conducted fresh embryo transfer and 148 women chose frozen embryo transfer, while 200 women opted for fresh embryo transfer and 79 women chose frozen embryo transfer in the non-surgery group. The implantation rate, clinical pregnancy rate, and LBR showed no significant difference between the two groups either in fresh embryo cycles or in frozen embryo transfer cycles, respectively.

**Table 5:** Pregnancy outcomes in endometrioma women with and without surgery.

Characteristics^a^	Endometrioma with surgery	Endometrioma without surgery	P
Fresh-ET			
Cycles	401	200	
No. of transferred embryos	1.91 ± 0.41	1.90 ± 0.43	0.675
Implantation rate (%)	39.82 (305/766)	39.05 (148/379)	0.803
Clinical pregnancy rate (%)	55.36 (222/401)	54.5 (109/200)	0.841
Live birth	36.91 (148/401)	38.00 (76/200)	0.387
Frozen–thawed ET			
cycles	148	79	
Implantation rate (%)	51.21 (127/248)	55.84 (86/154)	0.365
Clinical pregnancy rate (%)	33.78 (50/148)	40.50 (32/79)	0.315
live birth rate (%)	34.46 (51/148)	40.51 (32/79)	0.145

ET, embryo transfer; ^a^Continuous variable are expressed as mean ± standard deviation, SD, categorical variables as absolute frequencies, n (%). *P < 0.05 was considered statistically significant.

## Discussion

The results of this study demonstrated that the ovarian reserve and response following ovarian stimulation for IVF/ICSI treatment was significantly lower in patients with ovarian endometrioma compared with controls after adjusting for age, BMI, and infertility duration. Nevertheless, the pregnancy outcomes in cycles were comparable between the two groups. The proportions of top-quality embryos and available embryos did not differ significantly between the two groups. The lower CLBR was partly attributed to the decreased number of available embryos and top-quality embryos in endometrioma women, which was consistent with the findings of Boucret et al. (22). Surgical intervention prior to IVF/ICSI treatment in endometrioma women improved oocyte maturation and fertilization rates, but not pregnancy outcomes, in either fresh embryo cycles or in frozen embryo transfer cycles. Notably, there was not a statistical difference in the impact of surgery on AMH and AFC in patients with endometrioma.

In this study, women with endometrioma had a significantly lower ovarian reserve (AMH and AFC) compared to the control group, which was consistent with prior studies that showed a decreased ovarian reserve in women with endometrioma, regardless of whether they had any previous ovarian surgery (23–25). A number of studies have tried to elucidate the mechanisms by which an endometrioma hinders ovarian reserve. On one hand, some studies argued that by distorting the ovarian histology in women, endometriomas might be a detrimental factor to fertility. Maneschi et al. (26) found a decreased number of follicles in histological sections of the ovarian cortex surrounding the endometrioma, and Schubert et al. (27) also reported that follicle density was reduced in the cortex surrounding endometrioma compared to other types of cysts, proposing that endometrioma *per se* damaged the ovary. On the other hand, Kitajima et al. (9) found that ovaries affected by endometriomas present premature follicle recruitment, higher rates of atresia, and lower quality of remaining primordial follicles, which may be related to a local (intraovarian) inflammatory environment. The exact mechanism of endometrioma *per se* in decreasing the follicles remains unclear, so further research in this area is still needed.

Studies have reported that endometrioma surgery could damage the ovarian reserve through the unintentional removal of healthy ovarian tissue, thermal and devascularization injury, and postoperative inflammatory response (14, 25, 28). Some studies confirmed a significant decrease in ovarian reserve after the excision of endometrioma within 1 year; however, other studies assessing AMH levels up to 1 year after surgery revealed that this decrease could be temporary and gradually recover (28–31). A prospective study including 39 women reported that 50% of women had higher AMH levels 1 year after surgery than at 1 month after surgery (32). In the current study, a history of surgery in endometrioma women seemed not to affect the ovarian reserve. In the center, women with cysts larger than 6 cm were recommended for laparoscopic surgery management, and the duration between post operation and IVF/ICSI treatment varied from 3 months to 2 years, which may partly account for the insignificant differences of ovarian reserve in the two subgroups.

The results of this research also revealed a significantly lower OSI, oocyte maturation and fertilization rate, and number of embryos and top-quality embryos in women with endometriomas, which was in accordance with the results of several previous studies (16, 23, 24). In the current study, surgical removal of the endometrioma was prone to improving the OSI, maturation rate, and fertilization rate, indicating that the presence of endometrioma was a hazardous factor for the development of the surrounding follicles. Follicular fluid is an important microenvironment in oocyte growth and maturation. High concentrations of inflammatory cytokines, free iron, and oxidative stress markers, which are thought to disturb olliculogenesis and result in poor oocyte competence, were found in follicular fluid from women with endometrioma (33). Da Broi et al. (34) reported that follicular fluid from women with EMS could compromise nuclear maturation and the meiotic spindles of *in vitro* matured bovine oocytes. Besides, MII oocytes collected from EMS patients showed increased cortical granule loss and zona pellucida (ZP) hardening, which could affect embryo development (35). Furthermore, women without EMS using oocytes from donors with EMS had significantly lower implantation rates when compared with those who used oocytes from donors without EMS (36).

Nonetheless, this study failed to show any significant difference in pregnancy outcomes between women with endometrioma and the control group, regardless of whether the women had any ovarian surgical history. Although the CLBR was lower in endometrioma women, it was mainly attributed to the lower number of available embryos. The lower number of oocytes retrieved also resulted in the lower number of available and top-quality embryos in women with endometrioma. A recently published meta-analysis showed significantly lower clinical pregnancy rates but no difference in live birth rates (23). The impact of EMS on pregnancy following IVF remains controversial, with some studies reckoning a significant negative impact and still others arguing that there is no effect (16, 37, 38). Since the retrospective and monocentric design of this particular study lowers the power of the conclusions and potentially misestimates the significance of factors, future studies on women with unilateral endometriomas to examine and compare embryo development in the affected *vs*. the normal ovary are suggested to further identify the impact of endometrioma.

In conclusion, the findings from this study support that ovarian endometrioma negatively impacts oocyte quality and quantity, but not pregnancy outcomes, in women undergoing IVF/ICSI treatment. Surgical excision of endometrioma prior to IVF/ICSI can partly improve oocyte maturation and fertilization but not affect pregnancy outcomes and may adversely affect ovarian reserve.

## Data Availability Statement

The raw data supporting the conclusions of this article will be made available by the authors without undue reservation.

## Ethics Statement

All procedures performed in this study were in accordance with the ethical standards of the Ethics Committee of The Sun Yat-sen Memorial Hospital of China (SYSEC-KY-KS-2020-143). Written informed consent for participation was not required for this study in accordance with the national legislation and the institutional requirements.

## Author Contributions

QZ supervised the entire study, including the procedures, conception, design, and completion of the study data. HL revised the article. RY, JL, and XJ were responsible for the collection of data. YW contributed the data analysis and drafted the article. All authors contributed to the article and approved the submitted version.

## Funding

This work was supported by National Natural Science Foundation of China (grant numbers 81971332).

## Conflict of Interest

The authors declare that the research was conducted in the absence of any commercial or financial relationships that could be construed as a potential conflict of interest.

## Publisher’s Note

All claims expressed in this article are solely those of the authors and do not necessarily represent those of their affiliated organizations, or those of the publisher, the editors and the reviewers. Any product that may be evaluated in this article, or claim that may be made by its manufacturer, is not guaranteed or endorsed by the publisher.
